# Liuwei Dihuang formula ameliorates chronic stress-induced emotional and cognitive impairments in mice by elevating hippocampal O-GlcNAc modification

**DOI:** 10.3389/fnins.2023.1134176

**Published:** 2023-04-20

**Authors:** Yan Huang, Jianhui Wang, Feng Liu, Chenran Wang, Zhiyong Xiao, Wenxia Zhou

**Affiliations:** ^1^Nanjing University of Chinese Medicine, Nanjing, China; ^2^Beijing Institute of Pharmacology and Toxicology, Beijing, China; ^3^State Key Laboratory of Toxicology and Medical Countermeasures, Beijing, China

**Keywords:** O-GlcNAc, Liuwei Dihuang formula, gut microbiota, O-GlcNAcase, O-GlcNAc transferase

## Abstract

A substantial body of evidence has indicated that intracerebral O-linked N-acetyl-β-D-glucosamine (O-GlcNAc), a generalized post-translational modification, was emerging as an effective regulator of stress-induced emotional and cognitive impairments. Our previous studies showed that the Liuwei Dihuang formula (LW) significantly improved the emotional and cognitive dysfunctions in various types of stress mouse models. In the current study, we sought to determine the effects of LW on intracerebral O-GlcNAc levels in chronic unpredictable mild stress (CUMS) mice. The dynamic behavioral tests showed that anxiety- and depression-like behaviors and object recognition memory of CUMS mice were improved in a dose-dependent manner after LW treatment. Moreover, linear discriminate analysis (LEfSe) of genera abundance revealed a significant difference in microbiome among the study groups. LW showed a great impact on the relative abundance of these gut microbiota in CUMS mice and reinstated them to control mouse levels. We found that LW potentially altered the Uridine diphosphate-N-acetylglucosamine (UDP-GlcNAc) biosynthesis process, and the abundance of O-GlcNAcase (OGA) and O-GlcNAc transferase (OGT) in CUMS mice, which was inferred using PICRUSt analysis. We further verified advantageous changes in hippocampal O-GlcNAc modification of CUMS mice following LW administration, as well as changes in the levels of OGA and OGT. In summary, LW intervention increased the levels of hippocampal O-GlcNAc modification and ameliorated the emotional and cognitive impairments induced by chronic stress in CUMS mice. LW therefore could be considered a potential prophylactic and therapeutic agent for chronic stress.

## Introduction

Mounting evidence has proposed that the dynamic form of intracellular post-translational modification (PTM) of proteins, O-linked N-acetylglucosamine (O-GlcNAc), as a sensor of stress pathways in organismal cells and tissues (Fehl and Hanover, 2022). The O-GlcNAcylation is regulated by the “writer” O-GlcNAc transferase (OGT) and the “eraser” O-GlcNAcase (OGA), which catalyze the addition and hydrolyzation of the GlcNAc moiety to the proteins, respectively (Chatham et al., 2021). To date, this dynamic PTM has been shown to participate in the pathogenesis and pathological process of various stress-related diseases, ranging from tumors (Hanover et al., 2018) to diabetes (Hegyi et al., 2020) and neurodegeneration (Yin et al., 2022). Hence, a developing hypothesis is that any abnormal physiological state with changed stress levels has an O-GlcNAc component (Yang and Suh, 2014). O-GlcNAc cycling is essential in the central nervous system, which has a higher level of OGT than peripheral tissues (Okuyama and Marshall, 2003). The catalytic deficiency of O-GlcNAc transferase was reported to contribute to X-linked intellectual disability, which directly reveals the key role of O-GlcNAc events in the nervous system (Pravata et al., 2019; Meek et al., 2021). Increasing global levels of O-GlcNAc by modulating OGT and OGA activity induced neuroprotection (Yuzwa et al., 2012) and slowed neurodegeneration (Wang et al., 2016a). The O-GlcNAc deficiency or depletion, using a conditional knockout mouse model, led to diminishing the pool of adult neural stem/progenitor cells and consequently aberrant adult neurogenesis *in vivo* (Chen et al., 2021). Reduced neuron-specific O-GlcNAc in the hippocampus elicited a decrease in synaptic protein expression and alteration in excitatory synaptic function (Wheatley et al., 2019). These evidence reveals an essential role for O-GlcNAc in the function of the neuronal system.

Due to the regulatory function of O-GlcNAc in synapse underlying memory formation, alterations in O-GlcNAc levels affected cognitive capability. Increasing the global O-GlcNAc of type 2 diabetes mellitus subjects was positively correlated with better memory function (Huang R. et al., 2019). Sleep deprivation cognitive impairment was facilitated by a reduction in cerebral O-GlcNAc levels (Lee et al., 2020). The catalytically inactive OGA elicited defects in habituation behaviors, demonstrating the requisite of O-GlcNAc for cognitive function (Muha et al., 2020). Due to the strong association between impaired glucose metabolism and depressive disorder (Elias et al., 2022), the altered O-GlcNAc flux is also involved in emotional behaviors. The OGA heterozygous rodents exhibited antidepressant-like behaviors, by chronically elevating cerebral O-GlcNAc levels (Cho et al., 2020). While abnormal levels of O-GlcNAc in mice of cognitive impairment caused by the pathologies of Alzheimer’s disease (Kim et al., 2022), hyperglycemia (Shi et al., 2021), and sleep deprivation (Kim et al., 2021) have been demonstrated, however, the role of O-GlcNAc in contributing to changed emotional and cognitive phenotypes under chronic stress conditions has been largely overlooked and unknown.

Previous research has found that the hormones in the hypothalamus pituitary adrenal axis, neuronal remodeling, intracerebral metabolism, and gut microbiome were strongly associated with chronic stress-induced behavioral disturbances (Bollinger et al., 2022; Morella et al., 2022; Zhou R. et al., 2022; Huang et al., 2023). Additionally, emerging evidence has implicated that O-GlcNAc modification might serve as a stress sensor (Liu Y. et al., 2020). Moreover, the OGT secreted from *Legionella*, *Photorhabdus*, and *Clostridium perfringens* could promote the host‘s protein O-GlcNAcylation to modify cellular processes (Guttenberg et al., 2012; Lu et al., 2015). The *cp*OGA, a promiscuous microbial OGA, could remove O-GlcNAc from TGF-beta activated kinase 1 binding protein 1 (Pathak et al., 2012). In addition, gut bacterial secreted OGAs could protect rodents from ulcerative colitis by hydrolyzing O-GlcNAcylated proteins in the host (He et al., 2021). According to current studies, gut microbiota might affect the level of intracerebral O-GlcNAc through short-chain fatty acids (SCFAs), bile acids, and gut-brain-liver interaction (Cuervo-Zanatta et al., 2022; Hurley et al., 2022; Wang T. Y. et al., 2022). These studies revealed that in addition to being regulated by the host‘s hydrolase and transferase, the O-GlcNAcylated proteins in the host might be also controlled by bacterial-secreted OGTs and OGAs. However, it remains an open question whether manipulating gut microbial communities could regulate the levels of the host‘s protein O-GlcNAcylation and subsequent physiological functions.

Liuwei Dihuang formula (LW) is a classical traditional Chinese medicine consisting of six herbs (Zhou et al., 2016). In recent years, numerous studies have indicated that LW displayed ameliorative effects on the impairments of cognitive function induced by D-galactose (Liu B. et al., 2022), β-amyloid (Sangha et al., 2012), and senescence (Huang et al., 2012). Moreover, further studies demonstrate that the active fraction and monomers of LW could also alleviate cognitive dysfunction via diverse mechanisms, including hippocampal transcriptome (Wang et al., 2017b), N-glycan (Wang et al., 2017a), neuroendocrine-immune system (Wang et al., 2016c), as well as intestinal microbiome (Wang et al., 2019; Huang et al., 2022). While the crosstalk between gut microbiota and O-GlcNAcylation has been previously described (He et al., 2021), the ameliorative effect of LW on emotional and cognitive dysfunction associated with O-GlcNAc modification has yet to be explored. In this study, a chronic unpredictable mild stress (CUMS) mouse model was used to observe the effects of LW administration on the emotional and cognitive impairments, the alterations of community diversity and composition of gut microbiota, as well as on the O-GlcNAc-related biosynthesis pathways and enzymes, aiming to understand whether the amelioration of emotional and cognitive impairments by LW is related to the regulations of gut microbiota and hippocampal O-GlcNAc modifications.

## Materials and methods

### Animals and treatment

One hundred adult Male C57BL/6J mice (6–7 weeks-old) were used in the present study, which was purchased from SiPeiFu (Beijing) Biotechnology Co., Ltd. (Beijing, China) and maintained under specific pathogen-free conditions at the Laboratory Animal Center, Academy of Military Medicine Sciences. Behavioral experiments were conducted after 1 week of adaptive feeding. The mice were fed and drank freely and individually housed at room temperature (25 ± 1°C) with 55 ± 5% humidity and a 12 h light/dark cycle (lights on 7:00–19:00).

**FIGURE 1:**
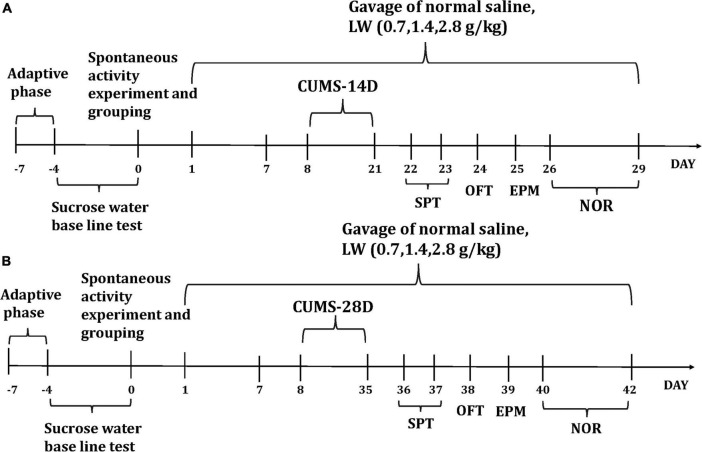
The experimental timeline and the effects of CUMS on mice. The dosage and days of intragastric administration and the specific time arrangement of each behavior after the end of CUMS-14D **(A)**, CUMS-28D **(B)**.

The experiment was assigned into two time periods of 14 and 28 days (Figure 1), each of which was subdivided into five groups, control group, chronic unpredictable mild stress (CUMS) group, low dose group (0.7 g/kg), middle dose group (1.4 g/kg), and high dose group (2.8 g/kg). All mice were randomly divided into five groups of 10 mice each. There was no stress or medication treatment in the control group. The CUMS group was given intragastric administration of normal saline. The other three groups were given different doses of the Liuwei Dihuang formula (0.7 g/kg, 1.4 g/kg, 2.8 g/kg). The LW (20071816, National Medicine Permission Number Z19993068, Beijing, China) was purchased from Beijing Tongrentang Technology Development Co., Ltd. The animal feeding environment and experimental program are following the relevant regulations of the Beijing Institute of Pharmacology and Toxicology and are approved by the Institute of Animal Care and Use Committee (IACUC) of the National Beijing Center for Drug Safety Evaluation and Research (NBCDSER).

### Chronic unpredictable mild stress

The modeling method of CUMS model is based on previous literature (Chevalier et al., 2020) with some modifications. In this study, 14 stress conditions were adopted, which were the following: food deprivation for 24 h, water deprivation for 24 h, cage tilt 45° for 24 h, daytime darkness for 12 h, overnight light for 12 h, wet padding for 24 h, no padding for 24 h, tail clamping for 2 min, restraint for 2 h, swimming at 4°C for 5 min, noise for 1 h (100 dB), foot shock for 1 h (1 mA, 5 s/min), tail suspension 30 min, cage vibration 1 h (220 r/min). Any two random combinations of stress styles per day.

### Behavioral assessments

#### Open field test (OFT)

The open-field test was used to measure activity and anxiety-like behaviors (Zhou L. et al., 2020). Thirty minutes before the test, the mice were put into the behavioral room for environmental adaptation, and then put into the center of the open field box of 40 cm × 40 cm × 40 cm (XR-XZ301, Shanghai Xinxin Information Technology Co., Ltd) and allowed to roam freely for 10 min. The whole experiment was recorded by the camera system (ANY-maze, Global Biotech Inc., USA), and the distance of the mouse movement in the central region within 10 min was recorded.

#### Elevated plus-maze test (EPM)

Each mouse was placed in the central position of the elevated cross maze (central area 5 cm × 5 cm, open arm 35 cm × 35 cm, closed arm 35 cm × 35 cm, and the height from the ground 76 cm, XR-XG201, Shanghai Xinxin Information Technology Co., Ltd.), and started facing the open arm area. Each mouse was tested for 5 min and recorded using animal behavior video (ANY-maze, Global Biotech Inc., USA) analysis software. The open-arm exploration time was used as an indicator of anxiety (Dong et al., 2020).

#### Sucrose preference test (SPT)

Before the formal start of the experiment (Liu et al., 2018a), the baseline of mice was tested first. The mice with a sucrose water preference rate of more than 80% were selected to ensure that the sugar water baseline of each group of mice was consistent, and then the SPT was conducted. The drinking bottle and squirrel cage specially used in the SPT was selected, and the water was first adapted to pure water for 24 h and then 2% sucrose water for 24 h under the same experimental environment. After the acclimation, all mice were deprived of water for 24 h, and a bottle of sucrose water and another bottle of pure water were placed above each mouse cage for 24 h test. After 12 h of testing, the positions of the two bottles of water were switched to exclude the effect of positional preference. The experiment was the same as the above details.

#### Novel object recognition test (NOR)

The novel object recognition test (Bevins and Besheer, 2006) was divided into three stages. The mice were put into a room 30 min before each stage for environmental adaptation. In the first stage of adaptation (days 1–2), mice were put into the testing box (XR-XX117, Shanghai Jilang Software Technology Co., Ltd) and allowed to freely explore for 20 min. After that, the box was wiped with 75% ethanol to avoid odor interference with the next mouse. In the second stage of learning (day 3), put two identical objects A and B into the box, and then put the mice on the side of the box wall away from the object, respectively. Each mouse was free to explore in the box for 16 min and recorded the total time T_*A*_, T_*B*_ of exploring the two objects. In the third stage of the test period (1 and 24 h after stage II), one end of the box was the original object A, and the other end replaced object B with a new object C. The test lasted for 4 min, and the total time (T_*A*_ and T_*C*_) of the mice exploring the original object A and the novel object C was recorded. The mice whose total exploration time of the two objects was less than 5 s were excluded. The preference index (PI) was used to evaluate the cognitive behavior of mice. The higher PI = T_*C*_/(T_*A*_ + T_*C*_), the better the recognition and memory ability of mice.

#### Western blot (WB)

Total protein was extracted from the hippocampus in RIPA lysis (C1053, APPLYGEN, China) buffer containing a protease inhibitor and supplemented with phosphatase inhibitors (11836170001, Roche, Germany), and centrifuged at 12,000 × g for 5 min at 4°C. The protein was quantified by the BCA protein assay kit. Protein lysates were cleared of insoluble material through centrifugation. Proteins were wet transferred to 0.2 μm pore size, hydrophobic PVDF transfer membranes (ISEQ00010, Millipore, Germany), which were blocked using 5% non-fat milk in 1% TBST buffer for 1 h at room temperature. The membranes were incubated overnight using the following primary antibodies: O-GlcNAc (CTD110.6, 1:1000 dilution) Mouse mAb (12938S, CST, USA), GAPDH (1:5000 dilution) Mouse McAb (60004-1, Proteintech, China). All primary antibodies were used in 5% BSA (CZ1006, czkwbio, China). Membranes were washed in 1%TBST (T1082, Solarbio, China) and incubated with the following appropriate secondary antibodies: goat anti-mouse HRP. The secondary antibodies were used at a 1:5,000 and 1:10000 dilution in 5% BSA. Protein bands were visualized following exposure of the membranes to ECL (RM0021, ABclonal, China) and quantified by densitometry analysis using Image software.

#### Enzyme-linked immunosorbent assay (ELISA)

The protein extracted from the hippocampus was brought to room temperature for ELISA. The manufacturer’s protocol for OGT (ml940027, Enzyme-linked Biotechnology, Shanghai, China) and OGA ELISA kit (ml950023, Enzyme-linked Biotechnology, Shanghai, China) was followed. Samples were visualized using an Enspire™ multilabel reader 2,300 (Perkin Elmer, Finland) at the 450 nm wavelength. The concentrations of OGT and OGA in the sample were calculated according to the standard curve, and the ratio of OGT to OGA is calculated.

### Mouse fecal collection and 16s rRNA sequencing

Fresh fecal samples (approximately 5–6 granules) from each mouse were collected and stored at −80°C for testing after the CUMS. Mouse fecal microbial genomic DNA was extracted according to the E.Z.N.A.^®^ soil DNA kit (Omega Bio-Tek, Norcross, USA) instructions, and the quality of DNA extraction was detected using 1% agarose gel electrophoresis. PCR products from the same sample were mixed and then recovered using 2% agarose Gel. AxyPrep DNA Gel Extraction Kit (Axygen Biosciences, Union City, USA) was used to purify the recovered products. The recovered products were detected by 2% agarose gel electrophoresis and quantified by Quantus Fluorometer (Promega, USA). Use NEXTFLEX Rapid DNA-Seq Kit to build libraries. Sequencing was performed using Illumina’s Miseq PE300/NovaSeq PE250 platform (Shanghai Majorbio Bio-pharm Technology Co., Ltd., China).

To study microbial diversity in the environment, the richness and diversity of the microbial community can be reflected by Alpha diversity analysis (Rogers et al., 2016; Zverev et al., 2021) in a single sample. Alpha diversity refers to the diversity in a specific region or ecosystem. Metrics in this study include sobs, chao, Shannon, and Simpson. By comparing species diversity in different habitats or microbial communities, Beta diversity analysis was conducted to explore the similarity or differences in community composition among different groups (Mohamed et al., 2021; Shen et al., 2021). Principal co-ordinates analysis (PCoA) is a non-binding data dimension reduction analysis method, which can be used to study the similarity or differences of sample community composition, PCoA was mapped based on the selected distance matrix, and was used to find out the potential principal components that affected the differences in sample community composition by dimensionality reduction. The differential gut microbiota obtain from the analysis of community composition (Ji et al., 2017) is based on data tables in the tax_summary_a folder and is graphed using R language tools. According to the results of taxonomic analysis, the species composition of different groups (or samples) at various taxonomic levels (such as domain, kingdom, phylum, class, order, family, genus, species, OTU, etc.) can be known. To evaluate the metabolic potential of the microbial community after LW intervention, the 16S rRNA sequence reading was clustered into operational taxonomic units (OTUs) using the closed reference method in QIIME2 software. Import the generated OTU table into PICRUSt (Langille et al., 2013; Tang et al., 2018; Ijoma et al., 2021), and use the Kyoto Encyclopedia of Genes and Genome (KEGG) database to predict the functional gene content of various microbial communities represented in the Greengenes 16S rRNA gene sequence database. In addition, for Pathway, PICRUSt was used to obtain information on three levels of metabolic pathways and the abundance tables of each level.

### Statistical analysis

GraphPad Prism 9.0 was used for graphing and statistical analyses. Student’s *t*-test was used for a two-group comparison of parametric data. One-way ANOVA with Dunnett’s or Tukey’s correction was used for multi-group non-parametric analyses. The behavioral data were combined by the *z*-score (Penny et al., 2019). A lower *z*-score signified an increase in behavioral deficits. The community richness of microbiota was analyzed by sobs and Chao, and the diversity of microbiota was analyzed by Shannon and Simpson. The microbial similarity was analyzed by principal coordinate analysis (PCoA). The effect sizes of the differentially abundant gut genus were analyzed by linear discriminant analysis (LDA) effect size (LEfSe) analysis (| LDA| > 2 and *P*-value < 0.05). Profiling of the predicted microorganisms was analyzed by phylogenetic investigation of communities by reconstruction of unobserved states (PICRUSt) method (Langille et al., 2013). The microbial data analysis was performed using the online platform Majorbio Cloud Platform.^1^ Data are depicted as mean ± S.D., with *P* < 0.05 considered statistically significant.

## Results

### LW ameliorates emotional and cognitive impairments of CUMS mice

**FIGURE 2:**
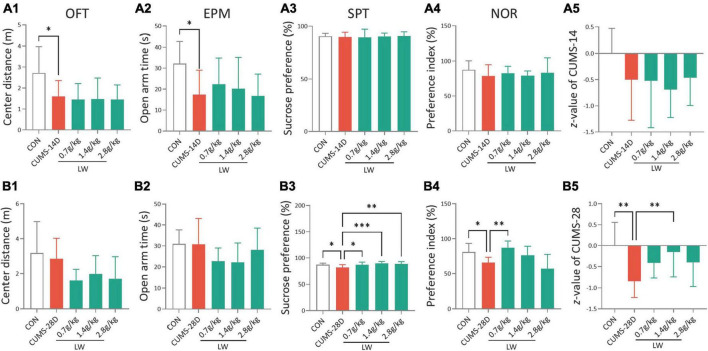
Liuwei Dihuang formula (LW) ameliorates the emotional and cognitive impairments of CUMS mice. Intragastric administration was started 7 days in advance and behavioral tests were performed after chronic stress. The distance in the center zone **(A1)** in the OFT, open arm time **(A2)** in the EPM, sucrose preference rate **(A3)** in SPT, preference index **(A4)** in the NOR test, and emotion and cognition z-score **(A5)** of mice on day 14 of CUMS. The center distance **(B1)**, open arm time **(B2)**, sucrose preference rate **(B3)**, preference index **(B4)**, and z-score **(B5)** of mice on day 28 of CUMS. *P < 0.05, ^**^P < 0.01, ^***^P < 0.001 were one-way ANOVA followed by Dunnett’s *post-hoc* test. The values are mean ± S.D., *n* = 10.

To investigate whether LW administration alleviates emotional and cognitive impairments induced by CUMS for 14 days in mice, the CUMS mice were fed with LW for 2 weeks. In the open field test and elevated plus-maze test, CUMS-14D mice exhibited significantly decreased center distance (Figure 2A1) and open arm time (Figure 2A2) than control mice. There were no group differences in sucrose preference rate (Figure 2A3), preference index (Figure 2A4), and *z*-score (Figure 2A5). However, the effects of LW (0.7, 1.4, 2.8 g/kg) on improving emotional and cognitive impairments were not obtained on day 14 of CUMS. These results indicate that the anxiety-like behavior appeared following 14-day CUMS but was unrelieved by administering three doses of LW.

For the 28-day CUMS, there were no group differences in the center distance (Figure 2B1) and open arm time (Figure 2B2). To examine depression in these mice, we employed the sucrose preference test (Figure 2B3). The CUMS-28D group exhibited a lower sucrose preference rate than the control group. Furthermore, administration of LW (0.7, 1.4, 2.8 g/kg) increased this rate in CUMS-28D mice. The results of the novel object recognition test (NOR) revealed a significantly decreased preferential index in CUMS-28D mice, and treatment with LW (0.7 g/kg) significantly improved the preferential index (Figure 2B4). The emotion-cognition phenotype evaluated by the composite *z*-score analysis showed a higher stress response of CUMS-28D mice in comparison to the control group, while 1.4 g/kg LW normalized the *z*-score in the CUMS group (Figure 2B5). These results indicated that the depression-like behavior and cognitive impairment appeared following 28-day CUMS and were relieved by administering 1.4 g/kg of LW.

### LW reshapes community diversity and composition of gut microbiota in CUMS mice

**FIGURE 3:**
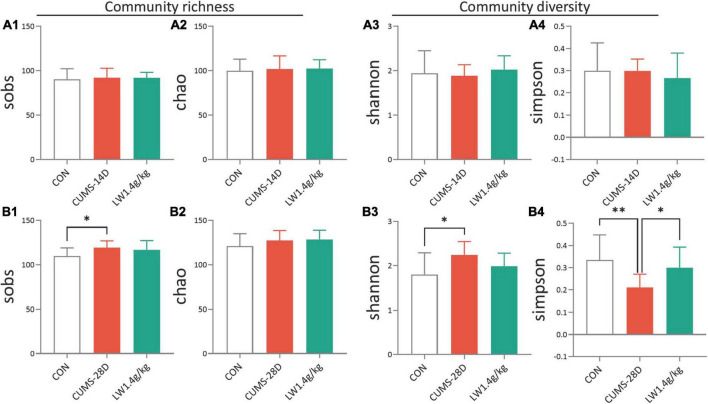
Liuwei Dihuang formula increases the community diversity of gut microbiota in CUMS mice. Mice to be sampled were placed in a clean cage covered with sterile filter paper after behavioral tests, and fecal samples were collected immediately after the mice defecated. This was used to detect gut microbiota. Different mouse samples should be replaced with new filter paper. The community richness of mice on days 14 **(A1,A2)** and 28 **(B1,B2)** of CUMS were analyzed by sobs and chao. The community diversity of mice on days 14 **(A3,A4)** and 28 **(B3,B4)** of CUMS were analyzed by Shannon and Simpson. *P < 0.05, ^**^P < 0.01 were one-way ANOVA followed by Dunnett’s *post-hoc* test. The values are mean ± S.D., *n* = 10.

Our previous studies have described that active fractions of LW restored cognitive deficits of adult senescence-accelerated mice by modifications of gut microbiota (Wang et al., 2016b,^2019^). Hence, we next examined whether LW (1.4 g/kg) intervention protection against CUMS-induced cognitive deficits was related to gut microbiome alterations. The composition and diversity of gut microbiota composition and diversity were analyzed by 16S sequencing. The results of alpha diversity analysis showed that there were no group differences in sobs (Figure 3A1) and chao (Figure 3A2) (richness of microbiota), or Shannon (Figure 3A3) and Simpson (Figure 3A4) (diversity of microbiota) on day 14 of CUMS, as shown in Supplementary Table 1. The CUMS-28D group exhibited higher sobs and Shannon, and a lower Simpson than the control group (Figures 3B1–B4), as shown in Supplementary Table 2. Moreover, the administration of LW normalized these indexes in CUMS-28D mice. These results showed that the prominently disordered microbiota richness and diversity were observed following 28-day CUMS. In terms of community diversity, we mainly tested Shannon and Simpson, LW administration improved the Shannon of CUMS mice and showed a trend of ameliorating the Simpson of CUMS mice.

**FIGURE 4:**
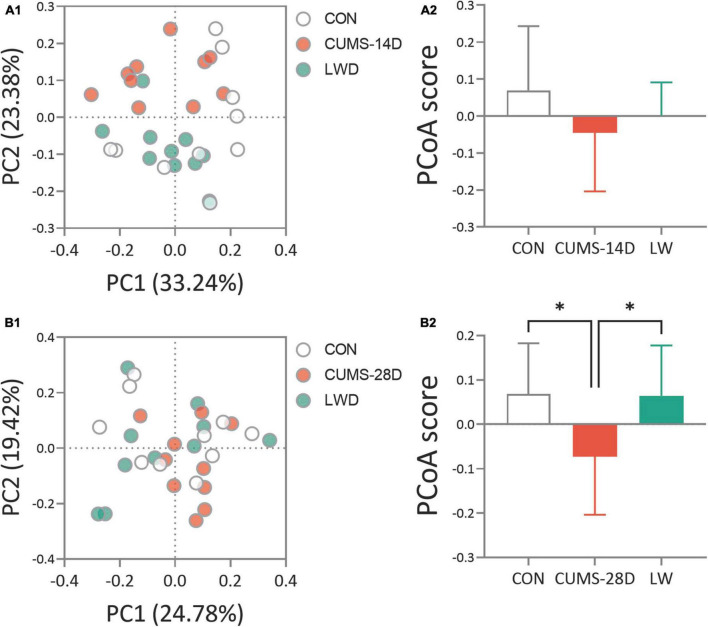
Liuwei Dihuang formula restored the beta diversity of gut microbiota in CUMS mice. After the basic collection of mouse feces, DNA extraction, and PCR amplification were performed to process the constructed Illumina Miseq sequencing data. Principle coordinate analysis (PCoA) plots were calculated by the Bray-Curtis similarity methods based on the microbial genus of CUMS-14D **(A1)**, CUMS-28D **(B1)** mice. Characterization of gut microbiota composition in CUMS-14D **(A2)**, CUMS-28D **(B2)** mice based on PCoA scores. *P < 0.05 was one-way ANOVA followed by Dunnett’s *post-hoc* test. The values are mean ± S.D., n = 10.

To analyze the dynamical similarities and differences of the gut microbiome in each group, principal coordinate analysis (PCoA) were performed. PCoA plot and score showed that there was no obvious separation between the three communities, control, CUMS-14D, and LW (Figures 4A1, A2 and Supplementary Tables 3, 4). In contrast, there was a significant separation of PCoA scores among groups on day 28 of CUMS (Figures 4B1, B2 and Supplementary Tables 5, 6). These data indicated that LW facilitated beneficial alterations in the structure of the gut microbial community, and the patterns of microbiota in the LW-treated group were more similar to the control group than the CUMS-28D group.

**FIGURE 5:**
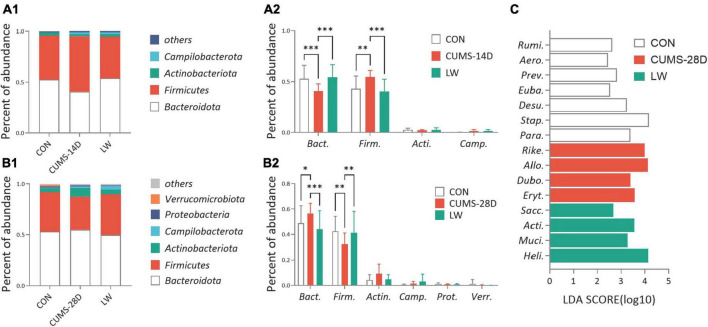
Liuwei Dihuang formula reversed the relative abundances of the predominant bacterial taxon of gut microbiota in CUMS mice. The average abundance of the predominant bacterial phylum (with relative abundance >1% in at least one sample) in CUMS-14D **(A1)** and -28D **(B1)** mice. Gut microbiota variations at the phylum level in CUMS-14D **(A2)** and -28D **(B2)** mice. **(C)** The linear discriminant analysis (LDA) effect size (LEfSe) classifying the most differentially abundant taxa at the genus level in CUMS-28D mice (| LDA| > 2 and *P*-value < 0.05 were shown). *P < 0.05, ^**^P < 0.01, ^***^P < 0.001 were one-way ANOVA followed by Dunnett’s *post-hoc* test. The values are mean ± S.D., *n* = 10.

To investigate the gut microbiota variations in response to LW intervention, we first compared the microbial composition at the phylum level (Figures 5A1, A2 and Supplementary Tables 7, 8). On day 14 of CUMS, the relative abundance of *Bacteroidota* phylum was significantly lower in CUMS-14D mice compared with control mice (52.51 vs. 40.75%), while the relative abundance of *Firmicutes* in the model group was significantly higher than that of the control group (43.38 vs. 54.65%), which were significantly restored by the LW intervention (Figure 5B1 and Supplementary Table 9). Conversely, CUMS-28D mice exhibited significantly increased *Bacteroidota* phylum (52.93 vs. 54.78%) and decreased *Firmicutes* phylum (38.79 vs. 32.60%) than control mice (Figure 5B2 and Supplementary Table 10). Next, we performed linear discriminant analysis (LDA) effect size (LEfSe) analysis (| LDA| > 2 and *P*-value < 0.05) to detect the dominant gut genus significantly affected by CUMS and LW treatment (Figure 5C and Supplementary Table 11). The relative abundances of the 7 genera, including *Rumi*. (*Ruminococcus_torques_group*, LDA = 2.63, *P* = 0.03), *Aero*. (*Aerococcus*, LDA = 2.45, *P* = 0.01), *Prev*. (*Prevotellaceae_NK3B31_group*, LDA = 2.82, *P* = 0.04), *Euba*. (*Eubacterium_nodatum_group*, LDA = 2.53, *P* = 0.01), *Desu*. (*Desulfovibrio*, LDA = 3.24, *P* = 0.02), *Stap*. (*Staphylococcus*, LDA = 4.17, *P* = 0.04), and *Para*. (*Parabacteroides*, LDA = 3.38, *P* = 0.01), were different in the control group. Moreover, 4 genera, including *Rike*. (*Rikenellaceae_RC9_gut_group*, LDA = 3.98, *P* = 0.0003), *Allo*. (*Allobaculum*, LDA = 4.13, *P* = 0.0005), *Dubo.* (*Dubosiella*, LDA = 3.38, *P* = 0.01), and *Eryt*. (*Erythrobacter*, LDA = 3.57, *P* = 0.04), were different in the CUMS group. When comparing the LW group with the control and CUMS group, 4 genera, including *Sacc*. (*Candidatus_Saccharimonas*, LDA = 2.68, *P* = 0.03), *Acti*. (*Candidatus_Actinomarina*, LDA = 3.55, *P* = 0.04), *Muci*. (*Mucispirillum*, LDA = 3.27, *P* = 0.02), and *Heli*. (*Helicobacter*, LDA = 4.14, *P* = 0.01), were revealed as characteristic gut genera.

### LW altered the O-GlcNAc-related biosynthesis pathways and enzymes of CUMS mice

**FIGURE 6:**
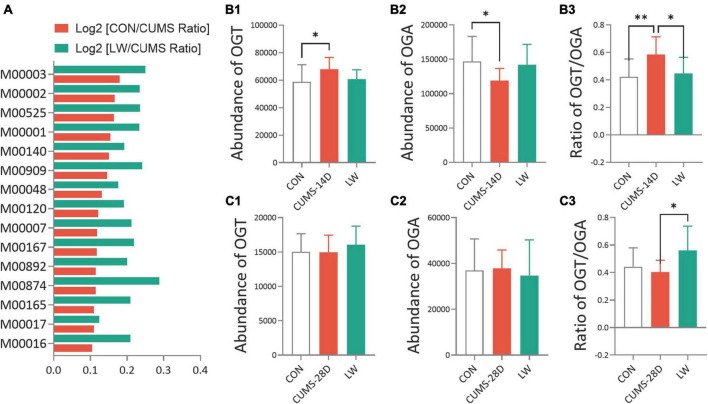
Liuwei Dihuang formula altered the O-GlcNAc-related biosynthesis pathways and enzymes of gut microbiota in CUMS mice. PICRUSt was used to standardize the OTU abundance table, that is, to remove the influence of the copy number of the 16S marker gene in the genome. Then, COG and KEGG functional annotations of OTU were carried out according to the corresponding green-gene id of each OTU, and annotation information of OTU at COG and KEGG functional levels and abundance information of each function in different samples were obtained. **(A)** The functional prediction of KEGG pathways using the phylogenetic investigation of communities by reconstruction of unobserved states (PICRUSt) based on OTUs of CUMS mice. Distribution of relative abundances of OGT **(B1,B2)**, OGA **(C1,C2)**, and the ratio of OGT/OGA **(B3,C3)** in CUMS-14D and -28D mice. *P < 0.05, ^**^P < 0.01 were one-way ANOVA followed by Dunnett’s *post-hoc* test. The values are mean ± S.D., *n* = 10.

To better understand the role of the gut microorganisms in CUMS and the potential underlying mechanism for LW ameliorating emotional and cognitive impairments, we then predicted functional pathway by the phylogenetic investigation of communities by reconstruction of unobserved states (PICRUSt) method (Figure 6A and Supplementary Table 12). The discrepancy in pathways relating to O-GlcNAc-related biosynthesis based on the KEGG database was observed when comparing the CUMS group to control and LW groups, including glycolysis (M00001 and M00002) (Nie et al., 2020), gluconeogenesis (M00003) (Misra et al., 2016), and UDP-N-acetyl-D-glucosamine biosynthesis (M00909 and M00892) (Umapathi et al., 2021). Then, the O-GlcNAc-related enzymes of the gut microorganisms after LW treatment were predicted (Figures 6B1–C3 and Supplementary Tables 13, 14). CUMS-14D mice exhibited a significantly increased abundance of OGT (Figure 6B1) and decreased abundance of OGA (Figure 6B2) than control mice. For the 28-day CUMS, there were no group differences in the abundance of OGT (Figure 6C1) and OGA (Figure 6C2). The ratio of OGT and OGA was positively associated with the level of O-GlcNAc modification. On day 14 of CUMS, the ratio of OGT and OGA was significantly higher in CUMS-14D mice compared with control mice and significantly decreased with LW administration (Figure 6B3). Within the 28-day CUMS, the ratio of OGT and OGA was no significant change but significantly increased by LW administration (Figure 6C3). The predictive functional analysis partly indicated that O-GlcNAc modification was one of the possible mechanisms of LW ameliorating stress-induced emotional and cognitive impairments.

### LW increased the hippocampal O-GlcNAc level and OGT/OGA ratio in CUMS mice

**FIGURE 7:**
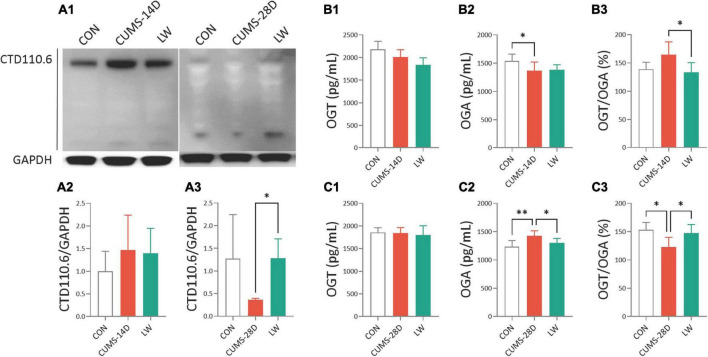
Liuwei Dihuang formula elevated hippocampal O-GlcNAc modification of CUMS mice. After behavioral tests, the mice were sacrificed, the hippocampus was isolated from the brain tissue and hippocampus protein was extracted. **(A1)** Representative Western blot of O-GlcNAc from whole hippocampal lysates of CUMS mice. Quantification of hippocampal O-GlcNAc modification levels in CUMS-14D **(A2)** and -28D **(A3)** mice, represented as mean optical intensity normalized to GAPDH. The concentrations of OGT **(B1,C1)**, OGA **(B2,C2)**, and the ratio of OGT/OGA **(B3,C3)** in the hippocampus of CUMS-14D and -28D mice were obtained by ELISA. *P < 0.05, ^**^P < 0.01 were one-way ANOVA followed by Dunnett’s *post-hoc* test. The values are mean ± S.D., *n* = 10.

To further examine the effect of LW on O-GlcNAc modification in CUMS mice, Western blot and ELISA were performed on whole hippocampal lysates (Figure 7). The uncropped blots can be found in Supplementary Material 1. There was also a trend to higher O-GlcNAc levels in the hippocampus of CUMS-14D mice (Figures 7A1, A2), and lower O-GlcNAc in CUMS-28D mice (Figures 7A1, A3), although the differences here were not statistically significant. LW treatment significantly increased hippocampal O-GlcNAc modification of CUMS-28D mice. There were no group differences in hippocampal OGT concentration in CUMS-14D (Figure 7B1) and -28D mice (Figure 7C1). For the 14-day CUMS, the hippocampal OGA concentration was observably decreased in CUMS group (Figure 7B2), and the ratio of OGT and OGA was significantly decreased by LW administration (Figure 7B3). For the 28-day CUMS, the hippocampal OGA concentration in CUMS group was significantly increased beyond the control group, while LW normalized this concentration in the CUMS group (Figure 7C2). The ratio of OGT and OGA was significantly decreased in the CUMS group and increased significantly with LW administration, which was consistent with the results of Western blot (Figure 7C3). Although there was no direct evidence that bacterial OGA or OGT could enter the brain, we found that there was a significantly positive correlation between the O-GlcNAc-related enzymes analyzed by PICRUSt and these assayed by ELISA (Supplementary Figure 1). These results indicated that LW intervention potentially decreased hippocampal OGA concentration and increased O-GlcNAc level by modifying the gut microbiota of CUMS mice.

## Discussion

**FIGURE 8:**
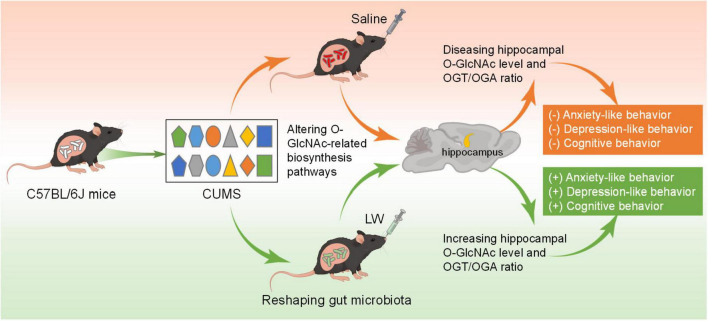
Sketch map for the mechanisms of LW on CUMS induced emotional disorders and cognitive impairment. LW decreases the ratio of OGT/OGA levels in CUMS-14D mice but increases the hippocampal ratio of OGT/OGA in CUMS-28D mice. LW intervention increased the levels of hippocampal O-GlcNAc modification and ameliorated the emotional and cognitive impairments induced by chronic stress in CUMS mice.

Our previous studies have revealed that LW and its active fractions improved cognitive and emotional dysfunctions in various animal models by modulating hippocampal neurogenesis, neurogenic microenvironment, neuroendocrine immunomodulation network, long-term potentiation, hippocampal transcriptome, and intestinal microbiome (Wang et al., 2016c,2017b,^2019^; Wei et al., 2021; Huang et al., 2022). Nevertheless, the role of O-GlcNAc modification in LW ameliorating emotional and cognitive function in CUMS mice, and the specific enzymes involved has generally received scant attention. Here, our results showed that anxiety-like behavior was present approximately at early-CUMS phase (day 14 of CUMS), and depression-like behavior and memory impairment were present on day 28 of CUMS. The administration of LW led to emotional and cognitive improvements in CUMS mice. Mechanistically, LW potentially modified the UDP-GlcNAc biosynthesis process, OGA and OGT levels by modulating the gut microbiome. Moreover, LW intervention increased hippocampal O-GlcNAc modification by elevating the ratio of OGT/OGA in the CUMS mice. These data revealed a significant role of O-GlcNAc modification in behavioral amelioration by LW intervention, which introduced a new mechanistic insight into the improved CUMS effects of LW on emotion and cognition (Figure 8).

To the best of our knowledge, the changes in anxiety-like behavior during the CUMS process were inconsistent in various studies. We have found that CUMS-14D center time was significantly different between the control group and the model group in OFT (Zhou X. D. et al., 2020). However, some literature also reported that there was no difference (Olave et al., 2022). And likewise, there are inconsistent results on day 28 of CUMS. There was significantly increased, decreased, or invariant total distance in the model group compared with the control group (Jeong et al., 2022; Wang C. et al., 2022; Zhou H. et al., 2022). Regarding the center time in OFT, there were also two opposite outcomes (Jeong et al., 2022; Wu et al., 2022; Zhou H. et al., 2022). Moreover, the center distance was not significantly different in OFT on days 28, 42, and 49 of CUMS (Liu et al., 2018c; Jiang et al., 2022; Wang G. et al., 2022), which was consistent with this study. We considered that the different experimental environments and conditions, and animal strains were the main possible reasons for the inconsistent results. And the indicators selected in this study were also relatively simple. In the further study, we will prolong the stress duration and repeat the experimental observation results to explore the deep mechanism of anxiety-like behavior appearing in CUMS-14D and disappearing in CUMS-28D.

Generally, stress can be divided into two forms: acute stress and chronic stress. Human studies have identified both acute and chronic stress as major risk factors for neuropsychiatric disorders, such as cognitive dysfunction (Zhang et al., 2021), major depressive disorder (Bernardo et al., 2022), anxiety disorder (Lopes Sakamoto et al., 2019), and Alzheimer’s disease (Muñoz-Mayorga et al., 2020). Recent studies over the span of the last decade showed the significant role of gut homeostasis in maintaining the host’s health during the stress process (Cruz-Pereira et al., 2020; Dawud et al., 2022). In this study, we focused on the dynamic changes in the distribution and composition of the gut microflora during chronic stress. We found that the dominant gut constituents in the LW-treated group were *Candidatus_Saccharimonas*, *Candidatus_Actinomarina*, *Mucispirillum*, and *Helicobacter* at the genus level. The *Candidatus Saccharimonas* microflora could be significantly elevated (Liu B. et al., 2022), and be related to inflammatory-related diseases (Cruz et al., 2020). Moreover, *Candidatus Saccharimonas* was a known short-chain fatty acid (SCFA) producer, and its increased abundance affected intestinal pH (Yao et al., 2022). The relative abundance of *Candidatus Actinomarina* was down-regulation in the chronic colitis model (Wu et al., 2020). Many studies have discovered that *Mucispirillum*, a Gram-negative, is highly connected with obesity, infection, inflammatory bowel disease, as well as stress (Anderson and Paschos, 2019; Herp et al., 2021). For example, the studies showed that the high abundance of *Mucispirillum* was associated with *Porphyromonas gingivalis*- and anesthesia/surgery-induced cognitive impairments (Chi et al., 2021; Lian et al., 2021). In addition, the elevated the relative abundance of *Mucispirillum* was correlated negatively with cognitive function in Alzheimer’s disease transgenic mice (Liu J. et al., 2020) and senile dementia mice (Gao et al., 2018). The *Helicobacter* is an endotoxin-producing microbe, which have a closely associated with ischemic stroke-induced cognitive impairment (Lv et al., 2022), and is implicated in the development of numerous cognitive-related disorders. For instance, increased *Helicobacter* was detected in chronic psychosocial stress, Parkinson’s disease and Alzheimer’s disease models (Cryan et al., 2019; Luo S. et al., 2022). All of these results suggested that the *Candidatus_Saccharimonas*, *Candidatus_Actinomarina*, *Mucispirillum*, and *Helicobacter* were essential for the improved effects of LW on CUMS mice.

To furtherly investigate the mechanisms underlying potential biological pathways of CUMS-induced emotional and cognitive impairments, PICRUSt based on the KEGG database was executed. Finally, we found that glycolysis, gluconeogenesis, and UDP-N-acetyl-D-glucosamine biosynthesis, which were particularly relevant to O-GlcNAc modification (Misra et al., 2016; Nie et al., 2020; Umapathi et al., 2021), might be the principal pathways in the differential flora. Hence, we assume that O-GlcNAc modification might play a significant role in CUMS. Various types of research have proved that dynamic protein O-GlcNAc modification is an intracellular signaling mechanism triggered by numerous stressors, including pressure-overload hypertrophy (Zhu et al., 2021), trauma-hemorrhage (Zou et al., 2007), cardiac injury (Jones et al., 2008), as well as a stress response (Fahie et al., 2022). As a stress receptor, O-GlcNAcylated p65 rapidly upregulated RNA binding motif protein 3 (Liu Y. et al., 2022) and interleukin-6 level (Hu et al., 2022) to maintain glucose metabolism and decrease apoptosis in the skeletal muscle of mice under acute cold exposure. Acute exercise stress significantly increased the O-GlcNAc level for cellular adaptation to oxidative stress, and this effect persists for at least 4 h (Peternelj et al., 2015). Additionally, etoposide (apoptotic agent), hydrogen peroxide, and glucose deprivation-induced a dynamic time-dependent O-GlcNAcylation in NCI-H1299 cells and Balb/c mice, and mutations at the S549 site (a major site of O-GlcNAcylation) of sirtuin 1 led to cell death (Han et al., 2017). The corticosterone release was mainly triggered by stress-induced activation of the hypothalamic-pituitary-adrenal (HPA) axis in rodents (Maggio and Segal, 2019). Corticosterone exposure for 60 h increased global O-GlcNac levels in female placentae (Pantaleon et al., 2017). Treatment of C2C12 with dexamethasone (a corticosteroid) for 48 h increased the O-GlcNAcylation level and concomitantly decreased protein phosphorylation level by reducing OGA expression at mRNA and protein levels (Massaccesi et al., 2016). Conversely, the long-term dexamethasone exposure (subcutaneously administered for 7 days) increased OGA expression concomitant with a decrease in OGT expression at protein levels (Liu et al., 2018b). In decoding the characteristic of O-GlcNAc in the stress process, the raising O-GlcNAc levels during acute stress was generally described as a cytoprotection effect, while the decreasing O-GlcNAc levels had cytotoxic effects, which is consistent with our present results. Our data have shown decreased OGA expression, increased ratio of OGT/OGA, and an increasing trend of O-GlcNAc levels in the hippocampus of mice at early-CUMS process. By contrast, hippocampal OGT/OGA and O-GlcNAc levels were decreased and the OGA level was increased at day 28 of CUMS. All of these results suggest that O-GlcNAc modification might be essential for relief from stress response in the CUMS process.

O-GlcNAc modification signaling serves as a sensor stress and nutrient to be involved in numerous manipulation of biological processes, which include gut microbiota (Dennis et al., 2006). The previous study has demonstrated O-GlcNAc down-regulation and dysfunction in patients with inflammatory bowel disease (Ma J. et al., 2022). Deficiency of OGT expression resulted in a damaged epithelial barrier and gut inflammation in mice, which were partially rescued by an OGA inhibitor (Zhao et al., 2020; Ma J. et al., 2022). Additionally, since bacteria-derived OGT and OGA have a similar catalytic domain as that of humans, they could modify host protein O-GlcNAc modification (He et al., 2021). However, there is currently no direct evidence to support bacterial OGA or OGT existed in the host’s brain. To the best of our knowledge, we did not find any direct or indirect correlation among these bacteria (*Candidatus_Saccharimonas, Candidatus_Actinomarina, Mucispirillum*, and *Helicobacter*) and OGA/OGT and O-GlcNAc. But we found a couple of flora that were associated with SCFAs (Wang et al., 2020; Gu et al., 2021; Liu Q. et al., 2021; Luo L. et al., 2022), glucose metabolism (Gao et al., 2020; Li L. et al., 2022; Luo L. et al., 2022; Ma Q. et al., 2022), glycolysis (Feichtinger et al., 2017; Zhou et al., 2021; Li L. et al., 2022), bile acids (Alizadeh and Raufman, 2022; Jian et al., 2022; Yin et al., 2022; Yuan et al., 2022) and other aspects. The intracerebral glucose metabolism and glycolysis, which produced the major donor substrate for O-GlcNAcylation (Liu X. et al., 2021), were regulated directly by intestinal microbiota and their metabolites. For instance, SCFAs were involved in the dysregulation of intracerebral glucose metabolism that occurs in the initial stages of Alzheimer’s disease (Zhang et al., 2022). Moreover, propionate promoted glycolysis in astrocytes of Alzheimer’s mice (Cuervo-Zanatta et al., 2022). Therefore, SCFAs could protect against brain-related diseases by preventing glucose metabolism (Rekha et al., 2022). In addition, bile acids synthesized in the gut could regulate cellular glucose metabolism in the brain (Hurley et al., 2022). Intestinal signals, which are transported to the brain, could also inhibit glucose synthesis via vagal afferent nerves (Xu et al., 2018; Bai et al., 2019). These results preliminarily showed that gut microbiota is one of the possible regulation manners in intracerebral O-GlcNAc modification. Recently, we found that the oligosaccharide fraction derived from LW reshaped gut microbiota and modified OGA abundance in the senescence-accelerated mouse (Wang et al., 2019). Hence, we sought to investigate whether LW administration changed O-GlcNAc modification of CUMS mice by gut microbiota, and the results confirmed our assumption. Here, in our work, we did observe LW treatment significantly elevated hippocampal O-GlcNAc levels in CUMS mice. Due to the O-GlcNAc modification being directly mediated by OGT and OGA, we next assayed the effect of LW on them. These preliminary results indicated that LW administration might improve the CUMS-induced emotional and cognitive impairments by increasing the levels of hippocampal O-GlcNAc and reducing OGA through gut microbiota. Currently, few researchers have investigated the effects of LW on O-GlcNAc. Our study represented the basis for future pharmacological research of LW and opened new perspectives in the discovery of prophylactic and therapeutic agents for chronic stress.

Our results further showed that LW decreases the ratio of OGT/OGA levels in CUMS-14D mice but increases the hippocampal ratio of OGT/OGA in CUMS-28D mice. It has been reported that changes in cell O-GlcNAc levels are affected by systemic physiological and pathological factors (Chatham et al., 2021). It has been proposed that OGT and OGA work together to form a “buffer” system to maintain the normal level of O-GlcNAcylation (Yang and Qian, 2017). LW-treated diseases were mainly connected with the restoration of the neuroendocrine immunomodulation (NIM) network (Huang Y. et al., 2019). The balance of the NIM network plays a key role in maintaining the physiological function of the body; thus, any imbalance in the NIM network is considered to be closely associated with diseases and the aging process (Besedovsky and Sorkin, 1977; Besedovsky and del Rey, 2002; Wang et al., 2018). It has been found that LW can rebalance the hypothalamic-pituitary-adrenal (HPA) and hypothalamic-pituitary-gonadal (HPG) axes and regulate the disturbance of the immune system and gut microbiota (Cheng et al., 2020). Our preliminary experimental results show that LW plays an integral role in regulating the balance of the NIM network by bidirectionally regulating the communication and interaction between the neuroendocrine and immune systems (Zhou et al., 2016). So LW plays a wide range of pharmacological roles by regulating and restoring NIM balance. The NIM network is interfered with by various pathological factors. The results of this study suggest that LW may balance NIM by regulating the change of O-GlcNAc levels.

There are still several limitations of this research. Firstly, in our work, we chiefly focus on the O-GlcNAc modification and did not devote attention to other types of protein glycosylation. The primary reasons are as follows. In recent years, over 5 thousand O-GlcNAcylated proteins have been identified (Ma et al., 2021) and implicated in multiple adaptive cellular processes (Ma J. et al., 2022). The latest authoritative research has revealed that O-GlcNAcylation effectively prevents cognitive declines (Xie et al., 2016), neurogenesis (Chen et al., 2021), and neural stem cell fate switch (White et al., 2020). The therapeutic effects of several traditional medicines might be related to normalizing the O-GlcNAc-modification (Diwu et al., 2013; Shi et al., 2021; Wu et al., 2021). The above advances in O-GlcNAc research enlighten our research future and idea in this work. Meanwhile, to the best of our knowledge, no other research is dealing with the effect of LW on O-GlcNAc modification so far. Secondly, to copiously uncover the specific O-GlcNAcylated protein involved in the physiological process of LW improving emotional and cognitive impairments, O-GlcNAcomic profiling was necessary. However, due to limited experimental conditions and resources, we did not complete the O-GlcNAcomic profiling in this study, which will be our future research priority. Thirdly, LW consists of 6 herbs: Dihuang (the prepared root of *Rehmannia glutinosa*), Shanyao (rhizome of *Dioscorea opposita*), Shanzhuyu (fruit of *Cornus officinalis*), Mudanpi (root bark of *Paeonia suffruticosa*), Zexie (rhizome of *Alisma plantago-aquatica*), and Fuling (sclerotia of *Poria cocos*). The composition and monomers of LW are detailed in this review (Zhou et al., 2016). Based on previous studies in our laboratory, the activity and active site of LW-AFC were mainly composed of polysaccharides, glycosides, and oligosaccharides (Huang Y. et al., 2019). We have done many studies on the principal component contribution of LW (Wang et al., 2016d,2017a,c; Huang Y. et al., 2019; Zeng et al., 2019; Cheng et al., 2020; Wei et al., 2021; Huang et al., 2022). We did not experimentally confirm the probable pharmacological effects of primarily active fractions or monomers in LW on O-GlcNAc modification, such as stachyose, paeoniflorin, morroniside, etc (Cheng et al., 2020). Although our insights provided a new perspective on the pharmacologic mechanisms of LW ameliorating stress-induced emotional and cognitive impairments, more precise and scientific experimental evidence is persuasive and integrity.

## Conclusion

Collectively, this study confirmed the capability of LW to ameliorate emotional and cognitive function, modulate gut microbial diversity and composition, and restored O-GlcNAc-related biological processes in CUMS mice. Moreover, this study, for the first time, demonstrated that the increased ratio of OGT/OGA and O-GlcNAc levels in the hippocampus induced by LW administration was one of the possible mechanisms of LW on CUMS mice, which deserves further investigation.

## Data availability statement

The datasets presented in this study can be found in online repositories. The names of the repository/repositories and accession number(s) can be found in the article/Supplementary material.

## Ethics statement

This animal study was reviewed and approved by the Institute of Animal Care and Use Committee (IACUC) of the National Beijing Center for Drug Safety Evaluation and Research (NBCDSER).

## Author contributions

WZ, ZX, and JW conceived the study, participated in its design and coordination, and helped to draft the manuscript. YH carried out the behavioral tests and wrote and revised the manuscript. FL and CW participated in the behavioral tests, biochemical analyses, and 16s rRNA analysis. All authors confirmed the final manuscript.

## Footnotes

1
www.majorbio.com

## Supplementary material

SUPPLEMENTARY FIGURE 1 **(A)** Correlation analysis (Pearson’s correlation) between the standardized abundance of OGT analyzed by PICRUSt and standardized hippocampal OGT concentration assayed by ELISA. **(B)** Correlation analysis between the abundance of OGA and hippocampal OGA. *n* = 5–6.
