# Supplementary figures and images for: Liuwei Dihuang formula ameliorates chronic stress-induced emotional and cognitive impairments in mice by elevating hippocampal O-GlcNAc modification
Source: Front Neurosci. 2023 Apr 20;17:1134176. doi: 10.3389/fnins.2023.1134176 (PMC10157057; doi:10.3389/fnins.2023.1134176)

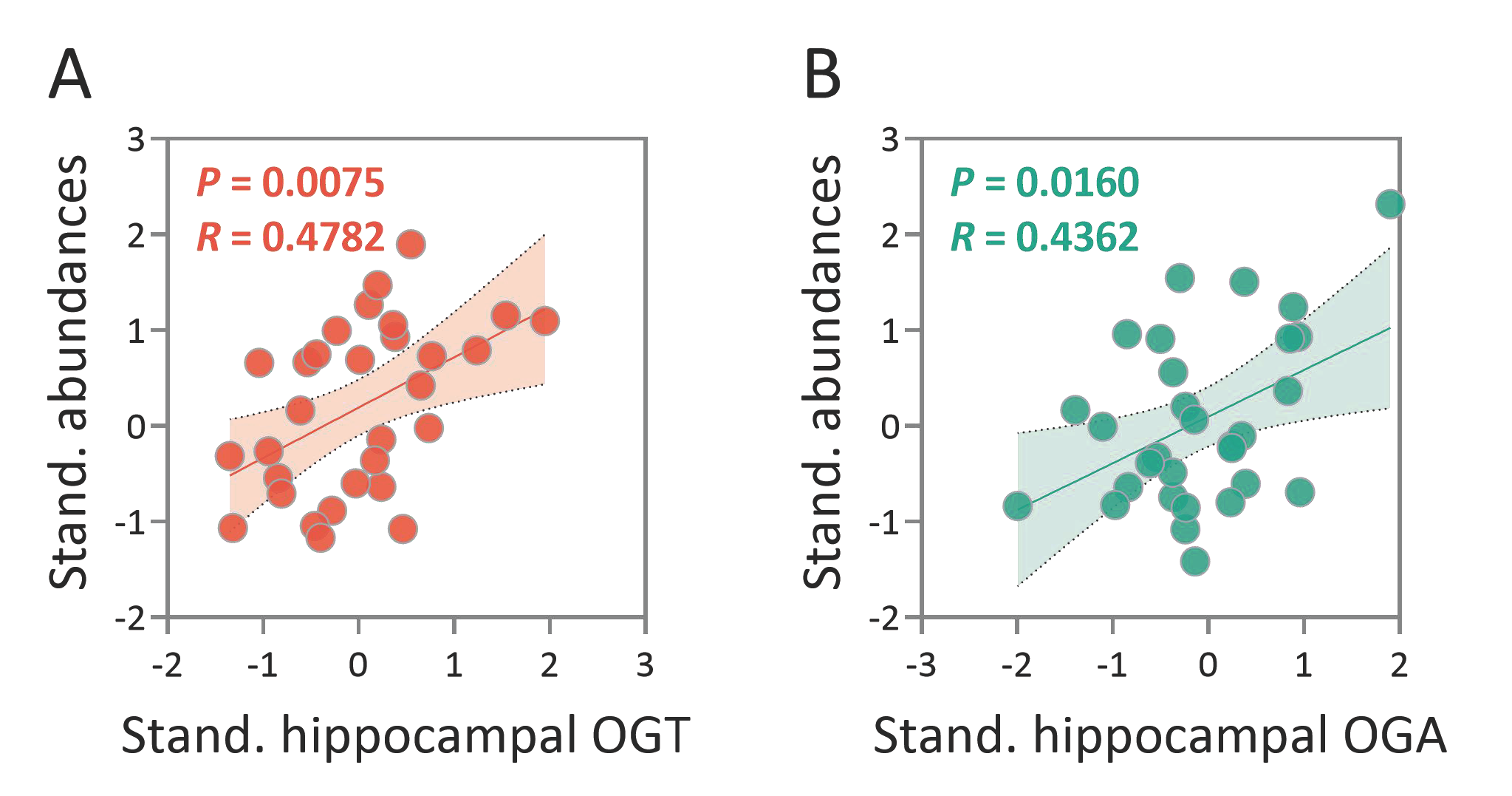

Supplement: SUPPLEMENTARY FIGURE 1 — (A) Correlation analysis (Pearson’s correlation) between the standardized abundance of OGT analyzed by PICRUSt and standardized hippocampal OGT concentration assayed by ELISA. (B) Correlation analysis between the abundance of OGA and hippocampal OGA. n = 5–6. [file Image_1.TIFF]
